# Maladaptive autonomic regulation in PTSD accelerates physiological aging

**DOI:** 10.3389/fpsyg.2014.01571

**Published:** 2015-01-21

**Authors:** John B. Williamson, Eric C. Porges, Damon G. Lamb, Stephen W. Porges

**Affiliations:** ^1^Brain Rehabilitation and Research Center, Malcom Randall Veterans Affairs Medical Center, Gainesville, FL, USA; ^2^Center for Neuropsychological Studies, Department of Neurology, University of Florida College of Medicine, Gainesville, FL, USA; ^3^Institute on Aging, Department of Aging and Geriatric Research, University of Florida, Gainesville, FL, USA; ^4^Department of Psychiatry, University of North Carolina at Chapel Hill, Durham, NC, USA

**Keywords:** PTSD, aging, polyvagal theory, chronic stress, autonomic nervous system

## Abstract

A core manifestation of post-traumatic stress disorder (PTSD) is a disconnection between physiological state and psychological or behavioral processes necessary to adequately respond to environmental demands. Patients with PTSD experience abnormal oscillations in autonomic states supporting either fight and flight behaviors or withdrawal, immobilization, and dissociation without an intervening “calm” state that would provide opportunities for positive social interactions. This defensive autonomic disposition is adaptive in dangerous and life threatening situations, but in the context of every-day life may lead to significant psychosocial distress and deteriorating social relationships. The perpetuation of these maladaptive autonomic responses may contribute to the development of comorbid mental health issues such as depression, loneliness, and hostility that further modify the nature of cardiovascular behavior in the context of internal and external stressors. Over time, changes in autonomic, endocrine, and immune function contribute to deteriorating health, which is potently expressed in brain dysfunction and cardiovascular disease. In this theoretical review paper, we present an overview of the literature on the chronic health effects of PTSD. We discuss the brain networks underlying PTSD in the context of autonomic efferent and afferent contributions and how disruption of these networks leads to poor health outcomes. Finally, we discuss treatment approaches based on our theoretical model of PTSD.

## INTRODUCTION

Post-traumatic stress disorder (PTSD) is a common diagnosis following trauma exposure. In a survey of 23,936 people with trauma exposure conducted by the World Health Organization World Mental Health Surveys across 13 countries, 6.6% evidenced clinical or subclinical PTSD symptoms consistent with DSM-V criteria (McLaughlin et al., 2014). In military samples, nearly 40% of people who experienced mild traumatic brain injuries developed symptoms of PTSD or depression (Tanielian et al., 2008).

Despite the prevalence of PTSD diagnoses, the consequences of trauma exposure, in particular in the context of PTSD, are far from deterministic. Exposure to trauma has varied outcomes between individuals, and repeated exposures may have different outcomes in the same person. At present, the underlying differences between individuals who appear to be resilient in the face of exposure to such trauma and those who are more likely to develop PTSD or another psychological disorder are not well understood. These individual differences in resilience and vulnerability are an important area of focus and may underlie chronic differences in health outcomes.

Current research is documenting the important mediating effects of several factors including genetics, developmental history, age, prior and present environment, and context. In this review, we will focus on the impact of chronic PTSD on the trajectory of health. Although PTSD is a categorical designation, there are varying degrees of severity of trauma-elicited perturbation of stress responses and disposition. Not everyone who is exposed to a traumatic experience develops symptoms that meet the categorical designation of PTSD. Furthermore, of those that do, many do not develop chronic dispositional shifts—i.e., they recover fully. Likewise, those that do not meet the criteria for a PTSD diagnosis may experience chronic effects of trauma exposure. In this review paper, we use “PTSD” as shorthand for PTSD continua symptoms as opposed to just the categorical designation.

The American Psychiatric Association (2013) recently revised the diagnostic criteria for PTSD [Diagnostic and Statistical Manual of Mental Disorders (5th Edition), 2013]. In order to be diagnosed with PTSD, one must have been exposed to one or more traumatic events that may include direct exposure, witnessing in person, indirect exposure, or repeated extreme indirect exposure to death, actual or threatened serious injury or actual or threatened sexual violence. The symptoms of PTSD are clustered into four categories: (1) intrusion symptoms (2) avoidance symptoms (3) negative alterations in cognition and mood and (4) alterations in arousal and reactivity [Diagnostic and Statistical Manual of Mental Disorders (5th Edition), 2013]. Related to these symptoms are shifts in autonomic state to deal with perceived threat. Specifically, the autonomic nervous system can shift state to efficiently promote mobilization for fight or flight behaviors or shift to a metabolically conservative state of quiescence that can manifest as extreme social withdrawal or even vasovagal syncope (i.e., fainting). Reaction to perceived threat takes precedence and displaces autonomic states that support social behavior, health, growth, and restoration. This automatic bias to prioritize potential (though often improbable, in the context of PTSD) threat is not entirely suppressed or easily modified by the higher cortical processes that we associate with voluntary behaviors, intentions, and learning. It is this loss of modulation of autonomic state that compromises a full range of functions from an ability to immobilize without fear in the presence of others, mobilize without rage or anger, and socially engage others. We conceptualize a loss of the range of autonomic state in response to social and environmental cues as a core component of PTSD itself (Williamson et al., 2013).

Chronic PTSD symptoms are related to several apparently disparate poor health outcomes including metabolic disease (e.g., possible increased diabetes risk; Miller-Archie et al., 2014), cardiovascular disease, asthma, cancer, back pain, peripheral vascular disease, gastrointestinal problems, thyroid disorders (Glaesmer et al., 2011) to mental health factors including loneliness (Kuwert et al., 2014) and hostility (Miller et al., 2013), which themselves are associated with physiological health consequences. However, through the lens of the autonomic nervous system, these disorders are related to an interaction between the autonomic nervous system and the regulation or modulation of myriad neurophysiological functions, from cognition to the immune responses. Below we discuss the contributions of autonomic dysregulation to deteriorating health. Within this context, we describe how autonomic functioning is related to central and peripheral inflammatory factors, immunologic functions, and psychosocial impairments (e.g., hostility and loneliness) in mediating and often exacerbating deleterious health outcomes.

## PTSD AND AUTONOMIC BEHAVIOR: CHRONIC THREAT AND THE PERPETUAL STATE OF FIGHT, FLIGHT, OR IMMOBILIZATION

Post-traumatic stress disorder manifests as a disorder across multiple systems in which there are atypical expressions of social engagement behaviors as well as disruptions in the regulation of emotional and cognitive processes. From an autonomic perspective, these processes and behaviors are consistent with a dispositional state of chronic mobilization or withdrawal in response to perceived threat. Affect, cognition, and autonomic functioning are interconnected via shared cerebral anatomy including cortical and subcortical structures and the white matter structures that connect them. The state of the autonomic nervous system is a component of nearly every function in which humans engage, including mobilization of cerebral hemodynamic resources to perform simple thinking tasks and mobilization of peripheral nervous system resources to engage with the environment, including through neurophysiological functions that involve muscle activity. The autonomic nervous system is integrated in all behavioral and physiological functions that are dependent on smooth, cardiac, and striated muscles.

Patients with PTSD process environmental stimuli differently than people without PTSD. For example, in a functional neuroimaging study comparing 23 patients with PTSD and 42 healthy controls, the patients with PTSD showed greater activation of the amygdala, a key limbic structure, in response to photographs of emotional facial expressions of fear (Felmingham et al., 2010). In another small study comparing 15 patients with PTSD and 15 age and sex matched controls, patients with PTSD demonstrated no ERP differences between processing of neutral and angry faces, suggesting reduced capacity to distinguish between threat and non-threat (Felmingham et al., 2003). Further, there is preliminary evidence that patients with PTSD have decreased high-frequency heart rate variability, likely indicative of an autonomic state that would support the mobilization necessary for fight or flight behaviors and resulting in lower vagal tone to the heart. This is perhaps due to the perception of a threat condition manifesting as a combination of increased sympathetic drive and/or parasympathetic withdrawal (Tan et al., 2011). Moreover, patients with PTSD also have altered acoustic startle responses, typically probed with brief but loud auditory stimuli. This alteration is not unimodal, as some people express greater physiological reactivity (increased startle reflex) whereas others, often those with severe or repeated trauma exposure, express physiological hyporeactivity (McTeague et al., 2010; D’Andrea et al., 2013).

Thus, PTSD may be understood as a deficit in autonomic adaptation that is often expressed as an incongruity between physiological state and environmental demands (Williamson et al., 2013). In the appropriate environment, a fight or flight state is adaptive. However, in environments that normally should be considered safe (e.g., sitting in an office or trying to sleep in one’s bedroom), such a state is maladaptive. When chronic, this incongruity may be pathological, even when only components of the mobilization response are chronic. For example, in Cushing’s syndrome there is a chronic state of elevated cortisol, and this disease is correlated with emotional and cognitive changes along with other health issues, including hypertension, diabetes, immunologic dysfunction, and sleep disturbances. As we will discuss, this is not unlike the health outcomes observed in patients with PTSD. Although an acute shift to a defensive physiological state can be an appropriate and effective response to environmental demands, a chronic fight or flight state is damaging. This damage includes metabolic, immunologic, and cardiovascular effects. Also, the psychological effects include impairment of close relationships with other humans. Thus, we propose that PTSD is associated with a pathological resetting of the autonomic nervous system that is manifest as an autonomic disposition to optimize defense reactions to danger and life threat.

## POLYVAGAL THEORY

Autonomic reactivity and the construct of autonomic disposition can be conceptualized within the framework of the Polyvagal Theory. The Polyvagal Theory, by providing a neurophysiological basis of how autonomic state and behavior interface, enables a better understanding of several behavioral and physiological features observed in PTSD. The theory emphasizes a hierarchical relation among three subsystems of the autonomic nervous system that support adaptive behaviors in response to the particular environmental features of safety, danger, and life threat (Porges, 1995, 2001, 2007, 2009).

The theory is named “Polyvagal” to emphasize that there are two vagal circuits. One is an ancient vagal circuit associated with defense. The second is an evolutionarily newer circuit, only observed in mammals, that is associated with physiological states related to feeling safe and spontaneous social behavior, and is important for social engagement. Consistent with this, potent regulators of our physiological state that mediate emotional expression are embedded in relationships (Cozolino, 2006; Siegel, 2012). Hofer (1994) employed a similar concept to explain the role of mother–infant interactions in facilitating the health and growth of infants. The core of the social engagement system in mammals is reflected in the bidirectional neural communication between the face and the viscera. Through reciprocal interactions via facial expressivity, gesture, and prosodic vocalizations, attunement occurs between the social engagement systems of two individuals. This attunement, consistent with Hofer’s insights, regulates behavioral states (i.e., emotional regulation), and simultaneously promotes health, growth, and restoration.

According to the Polyvagal Theory, when the individual feels safe two important features are expressed: first, bodily state is regulated in an efficient manner to promote growth and restoration. Functionally, this is accomplished through an increase in the influence of myelinated vagal efferent pathways on the cardiac pacemaker to slow heart rate, inhibit the fight–flight mechanisms of the sympathetic nervous system, dampen the stress response system of the hypothalamic–pituitary–adrenal (HPA) axis (e.g., cortisol), and reduce inflammation by modulating immune reactions (e.g., cytokines). Second, the brainstem nuclei that regulate the myelinated vagus are integrated with the nuclei that regulate the muscles of the face and head. This integration of neuroanatomical structures in the brainstem provide the neural pathways for a functional social engagement system characterized by a bidirectional coupling between bodily states and the spontaneous social engagement behaviors expressed in facial expressions and prosodic vocalizations (Porges, 2011; Stewart et al., 2013). Thus, the behavioral manifestation of this integrated social engagement system emerged specifically as a consequence of the neural pathways regulating visceral states becoming neuroanatomically and neurophysiologically linked with the neural pathways regulating the muscles controlling gaze, facial expression, head gesture, listening, and prosody (see Porges, 2001, 2007, 2009).

The autonomic subsystems responsible for defensive mobilization or restoration and social engagement can be conceptualized as phylogenetically ordered and behaviorally linked to three global adaptive domains of behavior including: (1) social communication (e.g., facial expression, vocalization, listening); (2) defensive strategies associated with mobilization (e.g., fight–flight behaviors); and (3) defensive immobilization (e.g., feigning death, vasovagal syncope, behavioral shutdown, and dissociation). These neuroanatomically based subsystems form a response hierarchy consistent with the construct of dissolution proposed by John Hughlings Jackson (Jackson, 1958) in which more recently evolved neural circuits regulate the function of older circuits. Therefore, the newest autonomic circuit associated with social communication has the functional capacity to inhibit the older involuntary circuits involved in defense strategies of fight/flight or shutdown behaviors.

Effective social communication is easier during states when we experience safety because our defense strategies are inhibited. This system is dependent on myelinated vagal pathways and fosters inhibition of sympathetic influences on autonomic behavior including dampening of the HPA axis (Bueno et al., 1989; O’Keane et al., 2005; Hostinar and Gunnar, 2013). Depending on when trauma occurs, there may be an interruption in the development of these social situations that impact cumulative life stress and health (Hostinar and Gunnar, 2013). Input to the nucleus ambiguus, which has direct output to the heart and bronchi via the newer myelinated vagus, promotes a state conducive to social engagement. As the dynamic process of threat detection increases, stage 2 (mobilization) or stage 3 (immobilization) responding occurs. Mobilization is characterized by sympathetic activation and parasympathetic withdrawal such that blood pressure increases and respiratory sinus arrhythmia (i.e., high-frequency heart rate variability) decreases. These physiological behaviors support high-energy actions including fighting or fleeing. Immobilization is an ancient defense strategy shared with most vertebrates and is facilitated by unmyelinated vagal pathways or “vegetative” vagus that slows the heart and inhibits subdiaphragmatic processes (e.g., digestion). This response strategy might be considered our most primitive defense system, and more severe trauma is associated with less physiological (i.e., sympathetic) reactivity and often complete collapse to threat stimuli (D’Andrea et al., 2013).

The Polyvagal theory has stimulated research across several disciplines (e.g., neonatology, obstetrics, bioengineering, pediatrics, psychiatry, psychology, exercise physiology, human factors, etc.) and has been used as a theoretical perspective to generate research questions and explain findings by numerous research teams (Travis and Wallace, 1997; Beauchaine, 2001; Beauchaine et al., 2007; Egizio et al., 2008; Hastings et al., 2008; Schwerdtfeger and Friedrich-Mai, 2009; Weinberg et al., 2009; Perry et al., 2012; Ardizzi et al., 2013). This theory has informed stress researchers of the important role the parasympathetic nervous system and its component vagal circuits play in neurophysiological mechanisms related to defensive strategies associated with reactivity, recovery, and resilience (Brown and Gerbarg, 2005; Kogan et al., 2012; Wolff et al., 2012; Evans et al., 2013; Kim and Yosipovitch, 2013).

## NEUROCEPTION

The Polyvagal Theory proposes a process, neuroception, which evaluates risk without awareness (Porges, 2003, 2007). Unlike perception, which involves a cognitive appraisal, neuroception involves brain processes that function outside the realm of awareness, although the individual may immediately become aware of consequences of these reactions when there are major shifts in autonomic state, such as palpitations, tachycardia, bradycardia, vasovagal syncope, nausea, or light headedness (Porges, 2003, 2007). Neuroception is supported by sensory and association cortices including temporal cortex, fusiform gyrus (face detection), and limbic structures including the amygdala (Adolphs, 2002; Pessoa et al., 2002; Winston et al., 2002) and orbitofrontal cortex (Grèzes et al., 2014). Neuroception is viewed as an adaptive mechanism that can either turn off or turn on defenses to engage others. Moreover, as this process triggers shifts in autonomic state, it may also bias perception of other people in a negative or positive direction. If our physiological state shifts toward behavioral shutdown and dissociation, mediated by the unmyelinated vagal pathways, we lose contact with the environment and others. The resulting observation in PTSD would be an autonomic disposition that would support defensive behavioral strategies.

Our nervous system continuously monitors and evaluates risk in the environment. When features of safety, danger, or life threat are detected, areas of the brainstem that regulate autonomic structures are activated. When features of safety are detected, autonomic reactions promote open receptivity with others, but when features of threat are detected, autonomic reactions promote a closed state limiting the awareness of others (Porges, 2003, 2007). For example, in the presence of someone with whom an individual feels safe, a person experiences the sequelae of positive social engagement behaviors consistent with a neuroception of safety; their physiology calms and their defenses are inhibited. Defensive strategies are then replaced with gestures associated with feeling safe and with this state of safety there is a perceptional bias toward the positive. Appropriately executed prosocial spontaneous interactions reduce psychological and physical distance. However, activating a sense of safety is greatly challenged in PTSD.

## RESETTING THE AUTONOMIC NERVOUS SYSTEM TO SUPPORT ADAPTIVE RESPONSES TO DANGER AND LIFE THREAT

Autonomic dispositions, or adaptive or maladaptive autonomic dynamic responses to external and internal environment cues, may be reset by trauma-related disruptions to the hierarchically organized neural regulation of the autonomic nervous system. This disruption may occur via physical trauma (e.g., TBI) injuring relevant systems (Williamson and Harrison, 2003; Smith-Bell et al., 2012), by genetics (Koenen, 2007; Glatt et al., 2013), or by a combination of factors, e.g., the diathesis-stress model (Edmondson et al., 2014).

Patients with PTSD may have a lower threshold to move from a neutral to a defensive state. PTSD alters threat detection such that information that may be identified as innocuous by someone who does not have PTSD (e.g., a car backfiring) is identified to be a threat by someone who does (e.g., a car backfiring is interpreted to be a gunshot). Their perception of threat is a manifestation of individually endogenous variables (personality, trauma history, sleep/arousal status, current transient stress levels, perceived control) and environmental variables (accessibility of exits, lighting levels, presence of strangers). Some aspects of PTSD may be a defensive strategy to environmental features that are outside of conscious awareness. For example, many people with PTSD, seemingly habitually, position themselves in a room with their back to a wall (i.e., no one can approach from behind), optimize access to an exit, and monitor visibility to directions of potential threat. To shift from this chronic defensive disposition, the patient with PTSD must be able to not only perceive, but to have an accurate neuroception of safety, which would inhibit limbic structures that mobilize defensive visceral states.

Though a person with PTSD may be able to consciously state that a given situation is safe, they may be unable to shift to the appropriate physiological state due to a disconnect between their cognitive appraisal and their bodily (e.g., autonomic) reactions. Perhaps an apt analogy is the difference between anosognosia and anosodiaphoria; in the former, the patient is unaware of a deficit, whereas in the latter, the patient is able to express the deficit, but cannot realize the emotional consequences of the issue. Both of these are more likely with right hemisphere lesions and there may be a right hemisphere bias in the process of neuroception such that sympathetic drive and mobilization defense strategies are supported preferentially by right hemisphere systems.

## CHRONIC STRESS, LONG TERM HEALTH CONSEQUENCES

The chronic stress associated with PTSD is a critical health issue as the physiological reaction to threat detection is metabolically costly. Although an invaluable survival tool in short-term, contextually appropriate situations, chronic engagement of threat response systems may lead to deterioration of health and social relationships, and ultimately, accelerate the aging process including decreasing age of onset of cognitive decline and early disability. Several features in the resting heart rate spectra (very low frequency, low frequency, and high frequency) are correlated with mortality (Williamson et al., 2010). In particular, low frequency heart rate variability is an independent predictor of death (Tsuji et al., 1994). Furthermore, reduced low frequency baseline (rest) heart rate variability is also linked to coronary artery disease (Kotecha et al., 2012), and lower nighttime heart rate variability as indexed by the standard deviation of RR internals is linked to increased stroke risk even in apparently healthy people (Binici et al., 2011). Allostatic load (McEwen and Wingfield, 2003) from incongruent threat detection and chronic defensive autonomic disposition is a chronic stress with cumulative consequences, however, it may be treatable. In a study of twenty PTSD discordant twin pairs (one having PTSD and one not), reduced heart rate variability was evident in the twins that had PTSD. Furthermore, patients who had recovered from PTSD (no longer reporting symptoms) were not different from the matched healthy twin in the heart rate variability measures. This suggests that at least a portion of the negative health consequences of PTSD may be reversible if treated early (Shah et al., 2013).

This is important because PTSD is associated with poor health outcomes. Patients with PTSD subsequent to the World Trade Center attacks have a tendency to develop diabetes (Miller-Archie et al., 2014) and have a higher likelihood of developing cardiovascular disease (Jordan et al., 2013). Also, in a sample of 52,095 people surveyed by the World Health Organization, patients with PTSD or other disorders of emotional regulation (e.g., depression) have an increased risk for developing cancer (O’Neill et al., 2014).

In addition to being associated with poor physical health outcomes, PTSD is associated with other mental illnesses and poor health behaviors, suggesting that health outcomes in this population are likely multifactorial. Miller et al. (2008) examined personality structure in patients with PTSD and found, using a structured clinical interview, that PTSD was best characterized by two internalizing factors, anxious-misery (correlating with PTSD and depression), and fear (correlating with panic disorder and obsessive compulsive disorder), along with one externalizing factor associated with antisocial personality disorder traits and substance abuse. The multifactorial basis of poor health outcomes is exemplified by PTSD’s association with alcohol dependence, as when PTSD is comorbid with a substance use disorder, it has been found to be the primary disorder in 60–85% of patients (Epstein et al., 1998). Even though PTSD is the primary disorder, alcohol dependence drives myriad poor health and social outcomes. In addition, patients with PTSD are more likely to smoke cigarettes (Gabert-Quillen et al., 2014; Lombardero et al., 2014), smoke at higher rates (Calhoun et al., 2011a) and show greater dependence (McClernon et al., 2005; Fu et al., 2007). It is not clear if the predisposition to substance use and abuse in the context of PTSD is driven by PTSD itself or if both are driven by shared underlying factors. In the case of cigarette use, for example, smoking rates are associated with a greater likelihood of developing PTSD possibly due to enhancement of memory (memory quality is associated with the development of PTSD) and/or differences in prefrontal-executive functions predisposing dysregulation of limbic circuitry after trauma. Smoking may exacerbate symptoms of PTSD as evidenced by enhanced acoustic startle in patients with PTSD after smoking (Calhoun et al., 2011b).

In addition to higher substance use and abuse rates, patients with PTSD have higher rates of aggression and anger, in particular amongst combat Veterans (Novaco and Chemtob, 2002). For example, a recent study examining the prevalence of intermittent explosive disorder in patients with trauma exposure (*n* = 232) reported that 24% met criteria for lifetime intermittent explosive disorder diagnosis and that PTSD severity was a significant predictor of intermittent explosive disorder diagnosis (Reardon et al., 2014). This violence can also manifest as hostility, a dispositional-like trait that may be characterized by cynical/hostile attributions, anger, and aggressive behaviors (Brummett et al., 1998). Hostility, as measured by personality scales such as the Cook-Medley Hostility Scale [Minnesota Multiphasic Personality Inventory (MMPI)-derived tool] is associated with cardiovascular disease and also with body mass index, waist-to-hip ratio, insulin resistance, lipid ratio, triglycerides, alcohol use, and smoking (Bunde and Suls, 2006). Anger/hostility is related to stress exposure (e.g., trauma), exaggerated autonomic reactivity to stress including cognitive (Williamson and Harrison, 2003) and pain stressors (Herridge et al., 2004), and reduced heart rate variability (Sloan et al., 2001).

Hostility, independent of PTSD, is related to loneliness. Even at an early age, lonely children are hypervigilant to social threat (Qualter et al., 2013), thus there is some counter co-morbidity to PTSD constellation symptoms. These co-morbid symptoms/traits rely on the same brain systems, supporting the idea that shifts in autonomic states impact aspects of mood/personality in a predictable manner and suggesting that intervention in these systems would likely affect all of those behaviors. Loneliness predicts reduced physical activity (Hawkley et al., 2009) and increased blood pressure in older adults (Hawkley et al., 2009, 2006). Thus, the driver of health outcomes after trauma is not necessarily the categorical presence of PTSD, but rather a reaction to trauma that perturbs the dynamic homeostasis of the social engagement system such that some aspect of chronic defensive disposition is elicited. That could be a constellation of symptoms that manifests primarily as anger, sadness, isolation or an interaction/fluctuation amongst these states and dispositions that results in a more severe presentation of symptoms, chronic stress, and deleterious health outcomes.

Patients with PTSD, relative to non-PTSD patients, have reduced heart rate variability in response to trauma cues, require an exaggerated recovery time after exposure (Norte et al., 2013), and have higher blood pressure (Paulus et al., 2013). Furthermore, chronic PTSD increases in catecholamines (e.g., epinephrine and norepinephrine) suggest increased sympathetic load in patients with PTSD (Lemieux and Coe, 1995). Norepinephrine enhances attention and memory formation and increased norepinephrine levels in cerebrospinal fluid are associated with the severity of presentation of symptoms of PTSD (Geracioti et al., 2001). Baseline levels of catecholamines due to trauma history may influence responses to stressors. For example, women with a history of abuse, in response to a mild physical challenge (1 mile stationary bike ride), demonstrated significantly greater reduction in parasympathetic tone than a control population (Dale et al., 2009). An investigation into plasma cortisol concentrations of rape victims revealed that those who reported a history of previous sexual trauma to a new assault did not respond with the same increase in plasma cortisol that first time victims did (Resnick et al., 1995). These findings suggest a direct relationship between stress responses, autonomic nervous system behavior, the manifestation of PTSD, and a mechanism of health decline. It should be noted that characterizing trauma presence and response is a challenge. Since the perception of an event as being traumatic depends not only on the physical features of the event but also on personal history and individual neurobiological differences, several sources of variance contribute to individual subject experiences.

Chronic stress, such as is present with PTSD and depression, has a negative impact on hippocampal characteristics in both animal models (Uno et al., 1989; Magariños and McEwen, 1995; Luo et al., 2014; Tse et al., 2014) and in humans (Childress et al., 2013). Furthermore, mood disorders such as PTSD and associated comorbidities (e.g., depression) are associated with hippocampal atrophy (McEwen and Sapolsky, 1995; Smith, 2005) as well as cognitive disorders such as dementia, as has been demonstrated in former prisoners of war with PTSD (Meziab et al., 2014). This may be in part due to shared neurobiological factors associated with the development of PTSD and dementia or other neuroanatomical changes associated with aging. For example, recent evidence suggests that the presence of apolipoprotein E4 allele (ApoE4), a risk factor for the development of Alzheimer’s disease, moderates the relationship between trauma exposure and the development of PTSD (Lyons et al., 2013). In animal models, ApoE4 also affects lipid factors that influence the development of atherosclerosis (Ewart et al., 2014).

In addition to neuroanatomical impacts, PTSD also negatively influences sleep. In a recent laboratory sleep study, 13 veterans with PTSD were compared to 17 trauma-exposed controls and 15 healthy controls (van Liempt et al., 2013). Patients with PTSD showed more frequent awakenings during both nights of the study compared to both control groups. Further, heart rate was higher in patients with PTSD compared to both control groups. Finally, adrenocorticotropic hormone and cortisol levels were inversely related to the presence of slow wave sleep (van Liempt et al., 2013). Patients with PTSD, in comparison to those with Panic Disorder and healthy controls, show lower respiratory sinus arrhythmia (Woodward et al., 2009), suggesting persistent vagal withdrawal during sleep. PTSD is also associated with low brain gamma-aminobutyric acid (GABA) levels and higher insomnia severity scores, which are correlated with lower GABA, and higher glutamate levels in parieto-occipital cortex (Meyerhoff et al., 2014). These results suggest that the chronic defensive dispositions of patients with PTSD manifest even during sleep. Poor sleep quality is an independent contributor to poor health outcomes including impairments in cognition (Aricò et al., 2010), white matter (Kumar et al., 2014), and cardiovascular disease factors independent of sleep apnea (Tosur et al., 2014).

The literature suggests that chronic throttling of social engagement systems such that defensive reactions are dominant results in poor health outcomes. The mechanisms of these outcomes are multifactorial and include damage from health behavior choices that may be attempts to self-regulate autonomic tone (e.g., stimulating nicotinic acetylcholine receptors by smoking cigarettes and modulating GABA receptors by drinking alcohol in large quantities), direct effects from chronic changes in cardiovascular behavior (e.g., decreased heart rate variability and increased blood pressure), and metabolic inflammatory responses as indicated by the presence of diabetes.

## TREATMENTS OF THE SOCIAL ENGAGEMENT SYSTEM AND EFFICACY IN PTSD

Numerous therapeutic strategies have been employed to treat PTSD utilizing different targets of the disordered system. These targets include cognitive schemas, behavioral techniques, relaxation strategies, and pharmacological interventions. Cognitive interventions include methods such as cognitive processing therapy (CPT), in which automatic thoughts are challenged via learning different thinking strategies. Behavioral techniques include exposure methods such as prolonged exposure therapy or flooding (Bluett et al., 2014). Relaxation strategies include interventions such as mindfulness-based stress reduction (MBSR) and paced breathing. Pharmacological interventions include propranolol and serotonin reuptake inhibitors, among others. Further, electrical stimulation and direct nerve interaction techniques are also available including stellate ganglion blockade and trigeminal nerve stimulation. All of these likely have an effect either directly or indirectly on threat detection and autonomic disposition. For the purposes of this review, we focus our discussion on the impact of treatment on modulation of the autonomic nervous system as this treatment outcome (reduction of chronic stress responses and facilitation of shift to a state conducive to social engagement) may be mechanistically the most germane to health outcomes and also appears to be a central component of the presentation of PTSD. These stress-modifying strategies can be broadly categorized as parasympathomimetic or sympatholytic.

There are few empirically supported psychotherapy treatments for PTSD. The details of these treatments are generally outside of the scope of the purpose of this review, however, the effects on the social engagement system are germane. Briefly, most empirically supported psychotherapies for PTSD have, at their core, an exposure component. Exposure-based therapies are commonly used for the treatment of anxiety disorders including PTSD, both with and without pharmacological adjuvants. The basic premise is that presentation of conditioned fear-eliciting stimuli without presence of an aversive event will result in extinction of the conditioned fear response. This type of approach is very effective with conditions such as specific-phobia. Exposure is more complicated in the case of PTSD and scaffolding has been created to deliver an effective extinction-based treatment paradigm. Empirically supported treatments include prolonged exposure and CPT. Eye movement desensitization and reprocessing (EMDR) does have empirical support for efficacy, but there are few data supporting the eye movement component. Unfortunately, there are scant published studies examining physiological efficacy of these psychological therapies or changes in attentional systems. Prolonged exposure therapy has been shown to improve symptoms of irritable bowel syndrome in a case study of a patient with PTSD (Weaver et al., 1998). Sympathetic arousal does impact GI function, thus this suggests the possible effectiveness of modifying the stress response (Weaver et al., 1998). EMDR has been shown to increase heart rate variability, again suggesting a possible mediating effect of vagal mechanisms in alleviating symptoms of PTSD (Sack et al., 2008). Additional research is needed to explore the impact of these treatments on the core symptoms of PTSD, specifically startle-responses, vigilance-attention behaviors, and baseline and task-dependent autonomic responses.

A second category of treatments that have demonstrated efficacy in the treatment of PTSD, or potential efficacy as shown in animal models, work via direct modulation of the autonomic nervous system. Within this domain of autonomic modulation, strategies fall broadly into two categories: sympatholytic (i.e., down-regulating sympathetic nervous system tone) and parasympathomimetic (i.e., up-regulating parasympathetic tone).

Sympatholytic approaches that directly dampen sympathetic reactivity are a promising route of therapeutic intervention. Beta blockers (e.g., propranolol), a class of compounds that are antagonists of β1- and β2-adrenergic receptors, inhibit the action of norepinephrine and epinephrine at these sites and have shown promise in both animal models and humans when administered during the acute phase of trauma, however, results have been mixed (Cahill et al., 1994; Vaiva et al., 2003; Hoge et al., 2012; Bailey et al., 2013). Capitalizing on the fact that norepinephrine levels tend to be higher in patients with PTSD, in a case study and a preliminary pilot study, Lipov et al. (2012) demonstrated the utility of stellate ganglion blockade in alleviating symptoms of PTSD. This method prevents lateralized sympathetic input from the stellate ganglion to the peripheral nervous system. However, there is evidence that the stellate ganglion blockade affects intracranial activity such that there may be increased risk of cerebral ischemia due to reduced blood flow of the non-blocked hemisphere (Kim et al., 2013a). The stellate ganglion blockade is a direct sympathetic nervous system intervention, however, some reports of decreased vagal tone measured by respiratory sinus arrhythmia (i.e., high-frequency heart rate variability) have been reported to occur concurrently (Fujiki et al., 1999), possibly suggesting that there may be a potential positive influence on the social engagement system.

Two parasympathomimetic approaches with promise include (MBSR; Kabat-Zinn, 1982) and Vagal nerve stimulation (VNS). VNS has been an FDA approved treatment for epilepsy since 1997 and treatment resistant depression since 2005. More recently it has shown promise for numerous other disorders including PTSD. At the moment, this human work has limited interpretability due to inadequate subject numbers and lack of control groups, but a pilot study did demonstrate a significant improvement in treatment resistant anxiety disorders (George et al., 2008). Rodent models of PTSD treated with extinction learning and VNS have yielded very encouraging results (Fanselow, 2013; Peña et al., 2013). Peña et al. (2013) demonstrated rodents trained on auditory fear conditioning showed rapid extinction of the conditioned response when the exposure training was done in conjunction with VNS. Mechanistically, they attributed this to a localized release of norepinephrine in limbic CNS structures, and VNS stimulation has been demonstrated to increase norepinephrine output in the amygdala (Hassert et al., 2004). Norepinephrine relased in limbic structures including the amygdala and hippocampus is thought to facilitate memory formation, and, in the case of PTSD, to facilitate new associations with stimuli used in extinction paradigms. On the other hand, data on the consequences of VNS stimulation on parasympathetic tone are sparse and somewhat contridictory. In children with epilepsy, for example, VNS stimulation caused a reduction in respiratory sinus arrhythmia (DeGiorgio et al., 2009). However, it may be the case that stimulation parameters in many of these patients were intense enough to directly stimulate vagal efferents, as respiration was dramatically reduced as well. It may also be the case that epileptic children, who are known to have atypical autonomic regulation, may be a unique population in their response to VNS. In a porcine model, right VNS resulted in an increase in parasympathetic tone and a decrease in low frequency heart rate variability, which is associated with decreased sympathetic tone. In canines who received 1 week of low level VNS, stellate ganglion nerve activity (sympathetic) was reduced (Shen et al., 2011).

Mindfulness-based stress reduction (Kabat-Zinn et al., 1992) was originally used to treat chronic pain, but has become a subject of much interest as a potential treatment for PTSD. The efficacy of MBSR for PTSD symptoms has been largely positive (Kimbrough et al., 2010; Dutton et al., 2013; Kearney et al., 2013; Omidi et al., 2013; Earley et al., 2014), though much of this research has suffered from design weaknesses such as a lack of active control groups, i.e., waitlist control groups are often used or, in some cases, no control at all. Despite this limitation, these findings are encouraging. MBSR borrows from both traditional and more contemporary practices including mindfulness meditation, body scan medication, and Iyengar Yoga. Two portions of this practice have explicit focus on breathing (i.e., mindfulness mediation and Iyengar Yoga). Both the mindfulness meditation and Iyengar Yoga components of MBSR are introduced to participants with a focus on one’s own breath (Kabat-Zinn, 1982). During these components, explicit instruction to slow ones breath is not given, however, it has been reported in multiple investigations that breath slows during the practice (Ditto et al., 2006; Ahani et al., 2014). Given the influence of slow breathing and especially expanding the duration of exhalation upon vagal afferents, it is not a surprise that increases in parasympathetic tone as indexed by respiratory sinus arrhythmia have been reported in a recent pilot study (Krygier et al., 2013). Yoga, as a standalone intervention not integrated into MBSR, has been reported to have efficacy as a PTSD treatment (Descilo et al., 2010). However, yoga is not a standardized practice and is administered via widely divergent procedures. For example, in a pilot study, Hatha Yoga and Iyengar Yoga, closely related styles that encourage attention to breathing, have been demonstrated to increase parasympathetic tone in healthy populations (Papp et al., 2013). On the other hand, other yoga interventions for PTSD have employed rapid breathing and hyperventilation, but those interventions failed to elicit an increase in parasympathetic tone (Telles et al., 2010). Given the heterogeneity of yoga procedures that have been employed for the treatment of PTSD, drawing conclusions of mechanism and efficacy for PTSD are difficult (see Kim et al., 2013b for review of mind-body practices in PTSD treatment, including Yoga). Although, interventions involving slow, paced breathing, either explicitly or implicitly, tend to enhance parasympathetic tone.

## CONCLUSION, PTSD AND ACCELERATED AGING

Post-traumatic stress disorder is heterogeneous and, while many people with PTSD often spontaneously recover, many others struggle with chronic symptoms. While there is variability in presentation, the core symptoms are well conceptualized as a disruption in the social engagement system such that threat detection is over-generalized and patients are chronically in a defensive autonomic disposition. This defensive autonomic disposition may support either mobilization (i.e., lower respiratory sinus arrhythmia, increased blood pressure, heightened cardiovascular reactivity to perceived-threat stimuli, and elongated recovery to baseline) or immobilization (i.e., blunted cardiovascular responses to emotional stimuli and a generalized profile of avoidance and apathy), or a fluctuation between these defensive strategies. These chronic defense responses appear to be related to deterioration in health as characterized by early morbidity and mortality, most commonly via cardiovascular disease. Further, a cascade of psychological and physiological responses influences these health outcomes such that poorer quality social interactions contribute to the deterioration of social support networks. This compounds the situation, functioning as a negative feedback loop, aggravating the chronic stress response and further impairing the ability of the person to recover.

Chronic disorders such as cardiovascular disease and diabetes increase in prevalence with age. Diseases of the vasculature including the heart and brain are primary age-related diseases (Corella and Ordovás, 2014). In healthy aging, there is a reduction in respiratory influences on heart rate variability (Nemati et al., 2013), and these are exacerbated by cardiovascular risk factors. Further, there is a link between decreased heart rate variability associated with stress and the risk for vascular disease (Borchini et al., 2014). Thus, the chronic stress from trauma in young adults as they age may accelerate the normal aging process in part by prematurely introducing heart dynamics associated with older age.

Together, the PTSD related cardiovascular, metabolic, and inflammatory systemic changes affect the body in a manner consistent with accelerated aging. Further, pre-trauma exposure characteristics that predict future diseases of aging (e.g., ApoE4 status), are associated with vulnerability to the development of PTSD. Several groups have hypothesized early Alzheimer’s disease onset due to chronic changes associated with PTSD (Weiner et al., 2013; Greenberg et al., 2014), and linkages between PTSD diagnosis and dementia have been demonstrated in large samples. For example, in a sample of more than 180,000 veterans, those that were diagnosed with PTSD were more than twice as likely to develop dementia (Yaffe et al., 2010). PTSD has been linked to many brain changes including structural and functional shifts in medial prefrontal cortex, amygdala and hippocampus, with some studies showing longitudinal changes (Uno et al., 1989; Magariños and McEwen, 1995; Smith, 2005; Bremner, 2007; Childress et al., 2013; Luo et al., 2014; Tse et al., 2014). These regions, in particular frontal-temporal structures, are also particularly vulnerable in normal aging; e.g., limbic white matter tracts are amongst the structures that deteriorate the earliest and fastest with aging. (de Groot et al., 2014). Further, these regions are primary areas of amyloid deposition in Alzheimer’s disease (Fjell et al., 2014).

Due to potential chronic effects of both PTSD and aging on limbic areas and their cortical mediators, there is a possibility of a progressive decline of social engagement systems such that delaying intervention may affect the viability of treatment effectiveness. In normal aging, resting respiratory sinus arrhythmia is lower with age (De Meersman and Stein, 2007; Capuana et al., 2012) and task-dependent autonomic response reflects heightened sympathetic response (Capuana et al., 2012). Paralleling this, young adult patients with PTSD show task-dependent differences in respiratory sinus arrhythmia response such that RSA does not change in response to cognitive challenge in comparison to healthy age matched adults (Sahar et al., 2001). However, if the autonomic dysregulation associated with PTSD is addressed, interrupting the cascading effect of metabolic, autonomic, neuro-immunologic, and health behavior interactions, then we can dramatically improve health outcomes and greatly improve quality of life in these individuals. In particular, studies of early intervention in both comparative and human patients with trauma exposure focusing on treatments with parasympathomimetic effects may yield the best effect sizes for attenuating or arresting the impact of trauma on the acceleration of the aging processes in these patients.

Overall, focus on early treatment of trauma victims is a public health concern with great potential for economic impact. Interventions that focus on supporting physiological states, thus providing a neural platform for spontaneous social engagement behaviors by optimizing autonomic regulation and functionally dampening defensive mobilization and immobilization strategies, should have the highest impact. As is the case with interventions focused on improving health trajectories with aging, the earlier the treatment, the greater the eventual effect is likely to be. Assessing which defensive strategy is dominant may be key to the selection of effective interventions. Research on efficacy of treatment should treat the category of PTSD as heterogeneous and should examine differential response based on personality factors in the expression of PTSD symptoms and dispositional differences in chronic stress profiles. Ideally, treatment should result in normalization of cardiac vagal tone, reduction of overgeneralization of or faulty neuroception, and facilitate social engagement responses. These treatment targets show the greatest promise for assuaging the acceleration of physiological aging in chronic PTSD.

### Conflict of Interest Statement

The authors declare that the research was conducted in the absence of any commercial or financial relationships that could be construed as a potential conflict of interest.
